# Pathogens isolated from clinical cases of urinary tract infection in dogs and their antibiogram

**DOI:** 10.14202/vetworld.2018.1037-1042

**Published:** 2018-08-02

**Authors:** Manisha Punia, Ashok Kumar, Gaurav Charaya, Tarun Kumar

**Affiliations:** 1 Department of Veterinary Medicine, Lala Lajpat Rai University of Veterinary and Animal Sciences, Hisar, Haryana, India; 2 Veterinary Clinical Complex, Lala Lajpat Rai University of Veterinary and Animal Sciences, Hisar, Haryana, India

**Keywords:** antibiogram, multidrug resistance, *Escherichia coli*, *Staphylococci*, *Streptococci*

## Abstract

**Aim:**

This study aims to determine the etiology of urinary tract infection (UTI) in dogs and to develop an antibiogram of organisms isolated.

**Materials and Methods:**

Urine samples were collected either through catheterization or cystocentesis from 35 dogs suspected of UTI admitted to VCC, LUVAS, Hisar. Bacteria were identified on the basis of cultural characteristics in 22 samples, and all the isolates were subjected to *in vitro* antimicrobial sensitivity testing.

**Results:**

The urine samples found positive for bacteria yielded pure colony growth in 77.27% and mixed growth in 22.73% samples, respectively. *Escherichia coli* (29.62%) and *Streptococcus* spp. (29.62%) were the most prevalent microorganisms followed by *Staphylococcus* spp. (22.22%), *Klebsiella* spp. (11.11%), *Pseudomonas* spp. (3.7%), and *Bacillus* spp. (3.7%). Overall, maximum sensitivity of isolates was found toward ceftriaxone/tazobactam (88.88%) and least toward amoxicillin and cloxacillin (29.62%).

**Conclusion:**

*E. coli and Streptococcus* spp. were the most predominant bacteria isolated from UTI affected dogs. *In vitro* sensitivity revealed a significant proportion of bacteria to be multidrug resistant.

## Introduction

Urinary tract infection (UTI) refers to the microbial colonization of the urinary tract or of any urinary tract organ, except the distal urethra, which has a normal bacterial flora. Various microorganisms have been involved in the etiology of UTI in dogs. Approximately, 14% of all dogs encounter at least one episode of bacterial UTI during their lifetime [1]. Among bacterial causes, *Escherichia coli* has been the most frequently isolated bacteria causing UTI in dogs which can go up to 30% [2-4]. Other commonly isolated bacteria include *Staphylococcus* spp., *Enterococcus* spp., *Proteus* spp., and *Klebsiella* spp. [2-5].

Microbiological culture combined with susceptibility testing is the cornerstone of UTI diagnosis and the best instrument for guiding treatment decisions in individual dogs [6,7]. Periodic monitoring of pathogens isolated from UTI and their susceptibility patterns help in the choice of first-line empirical therapy and can also be used to monitor the presence of resistant bacteria.

Treatment with antibiotics is one of the most critical components to control UTI. Increasing antimicrobial resistance in canines is of concern as it complicates therapy in dogs, contributes to therapeutic failure, increased patient morbidity and mortality, and health-care costs in UTI infection. In addition, it is also a public health concern being pathogens/diseases zoonotic [8,9]. Therefore, definitive diagnosis of etiological agents and their antimicrobial sensitivity before treatment will help in selecting suitable and cost-effective antibiotic to treat the affected animal timely and adequately.

Therefore, the present study was aimed to determine the etiological agent(s) responsible for causation of UTI in dogs and to determine their antimicrobial sensitivity to institute proper line of treatment.

## Materials and Methods

### Ethical approval

The samples used in the current study were received from the clinical cases. As per University rule, approval of Institutional Animal Ethics Committee is not required for the clinical cases.

### Sample collection

A total of 35 dogs admitted to the VCC, LUVAS, Hisar, showing clinical signs suggestive of UTI such as inappetence/anorexia, emaciation, vomition, hematuria, polyuria, polydipsia, depression, weight loss, weakness, dehydration, nausea, anuria, stranguria, and oliguria were considered in the present study. Urine samples were collected using catheterization or cystocentesis. Of the 35 samples, 22 samples were collected by catheterization and 13 samples by cystocentesis.

### Bacterial isolation

Urine samples from the affected animals were collected aseptically in a sterile container. After receiving, the urine samples were inoculated in 0.01 ml volume on 5% sheep blood agar (BA) and MacConkey lactose agar (MLA) plates with the help of a 4 mm diameter platinum loop. The plates were incubated aerobically at 37°C for 24-48 h. Subcultures of the resulting growth were made on BA for purification of isolates and identified on the basis of Gram’s reaction, morphology, and colony characteristics. The Gram-positive cocci were subjected to catalase test to differentiate between staphylococci and streptococci. To rule out the possibility of micrococci, all the catalase-positive cocci were subjected to oxidase test and oxidation fermentation test. Other organisms such as *E. coli* and *Klebsiella* spp. were differentiated on the basis of growth on MLA and eosin methylene blue agar. Butyrous large colonies and no growth on eosin methylene blue were taken as *Klebsiella* spp., moreover, small shiny colonies with typical metallic sheen growth were taken as *E. coli*.

### In vitro antibiotic sensitivity

Different strains of various organisms isolated from urine samples of the infected animals were subjected to *in vitro* drug sensitivity testing, using 15 antimicrobials by the disc-diffusion method. With the help of a platinum loop, a small amount of test culture was transferred into a tube of brain heart infusion broth and incubated for 2-5 h at 37°C, to obtain turbidity. With the help of a sterile cotton swab, the broth culture was then evenly spread by smearing over the surface of BA/Mueller-Hinton agar plates. The antimicrobial discs were placed on the agar and gently pressed. These were then incubated at 37°C for 24 h. The sensitivity was observed on the basis of zone size interpretation chart, provided by the manufacturer. Different antimicrobials used were amikacin (10 mcg), amoxicillin (10 mcg), amoxicillin-clavulanic acid (30 mcg), ampicillin (25 mcg), cefoperazone (75 mcg), ceftriaxone (10 mcg), ceftriaxone–tazobactam (30mcg), chloramphenicol (25 mcg), cloxacillin (30 mcg), enrofloxacin (10 mcg), gentamicin (30 mcg), neomycin (30 mcg), oxytetracycline (30 mcg), penicillin-G (10 units), and streptomycin (25 mcg) (Hi-Media). To remain conservative in our estimates of resistance, isolates exhibiting intermediate zones of inhibition were interpreted as resistant.

### Determination of multidrug-resistant (MDR) bacteria

On the basis of sensitivity pattern, isolates were categorized into MDR, extreme drug resistant (XDR), and pandrug-resistant. Isolates resistant to three or more antibiotics belonging to different groups were classified as MDR. Among MDR isolates, isolates susceptible to only two antibiotics belonging to two different groups were considered XDR, while resistance to all the antibiotics was considered as pandrug-resistant.

### Statistical analysis

Results of overall antimicrobial sensitivity were statistically analyzed for the determination of confidence interval of percent number and Z test to reveal any significant difference between the different groups of antibiotics.

## Results

### Bacteriological examination

Of the 35 samples, 22 urine samples were found positive for bacterial isolation, of which 22 samples, 17 (77.27%) samples yielded pure culture, whereas mixed growth was observed in 5 (22.72%). Single isolation of *E*. *coli* was isolated in 5 (22.7%), *Streptococcus* spp. in 5 (22.7%), *Staphylococcus* spp. in 4 (18.18%), *Klebsiella* spp. in 2 (9.09%), and *Bacillus* spp. in 1 (4.54%). Among mixed cultures, one sample each was found positive for combination of *Streptococci + Klebsiella*, *Streptococci+ E. coli*, *Staphylococcus+ E. coli*, *Staphylococcus+ Streptococcus*, and *Pseudomonas+ E. coli*. A total of 27 isolates were recovered from urine samples. The frequency of isolation of different bacterial species is depicted in Table-1.

**Table-1:** Relative frequency of organism isolated from urine samples of dogs suffering from urinary tract infections.

Bacterial isolates	Number of samples positive	Percent positivity
*Streptococcus* spp.	5	22.7
*E. coli*	5	22.7
*Staphylococcus* spp.	4	18.18
*Klebsiella* spp.	2	9.09
*Bacillus* spp.	1	4.54
Mixed infections*	5	22.7

* *Streptococci+Klebsiella* (1), *Streptococci+Escherichiacoli* (1), *Staphylococcus+Escherichia coli* (1), *Staphylococcus+Streptococcus * (1), *Pseudomonas+Escherichia coli* (1)

### Antimicrobial sensitivity testing

Overall, maximum sensitivity of isolates was found toward ceftriaxone/tazobactam combination (88.88%) followed by ceftriaxone (77.77%), chloramphenicol (74.07%), gentamicin and cefoperazone (70.30%), amoxicillin/clavulanic acid and neomycin (55.55%), amikacin (48.14%), streptomycin (44.44%), ampicillin (40.74%), enrofloxacin (37.03%), oxytetracycline and penicillin-G (33.33%), and amoxicillin and cloxacillin (29.62%) as shown in Table-2. Statistically, Z test was applied to reveal any significant difference among the antibiotic used for *in* *vitro* sensitivity. Cephalosporin and macrolide group of antibiotics were found to be significantly sensitive as compared to penicillin group, whereas non-significant difference existed in sensitivity of tetracycline and penicillin groups of antibiotics. Among aminoglycosides group, gentamicin was found to be significantly sensitive compared to streptomycin but not with amikacin and neomycin.

**Table-2:** Antimicrobial sensitivity pattern of different bacterial isolates from urine of dogs afflicted with UTI.

Antimicrobials used	Sensitivity (%)	95% CI
Tetracyclines		
Oxytetracycline	9 (33.33)	15.55-51.11
Penicillins		
Ampicillin	11 (40.74)	22.21-59.27
Amoxicillin	8 (29.62)	12.41-46.85
Cloxacillin	8 (29.62)	12.41-46.85
Penicillin	9 (33.33)	15.55-51.11
Amoxicillin/clavulanic acid	15 (55.55)	36.81-74.30
Fluoroquinolones		
Enrofloxacin	10 (37.03)	18.82-55.25
Aminoglycosides		
Gentamicin	19 (70.3)	53.15-87.59
Amikacin	13 (48.14)	29.30-67.00
Neomycin	15 (55.55)	36.81-74.30
Streptomycin	12 (44.44)	25.70-63.19
Cephalosporins		
Ceftriaxone	21 (77.77)	62.10-93.46
Cefoperazone	19 (70.3)	53.15-87.59
Ceftriaxone/tazobactam	24 (88.88)	77.03-100.74
Macrolides		
Chloramphenicol	20 (74.07)	57.54-90.60

UTI=Urinary tract infection, CI=Confidence interval

Antibiotic sensitivity against various microorganisms isolated has been depicted in Table-3. *E*. *coli was* found most sensitive to neomycin (87.5%) followed by ceftriaxone-tazobactam, chloramphenicol, amikacin and gentamicin (75%), ceftriaxone and cefoperazone (62.5%), streptomycin (50%), amoxicillin-clavulanic acid (37.5%), and oxytetracycline and enrofloxacin (25%).

**Table-3:** *In vitro* antibiotic sensitivity pattern of different bacterial isolates.

Antimicrobials used	Sensitivity (%)					

	*E. coli* n=8 (%)	*Streptococcus* spp. n=8 (%)	*Staphylococcus* spp. n=6 (%)	*Klebsiella* spp. n=3 (%)	*Pseudomonas* spp. n=1 (%)	*Bacillus* spp. n=1 (%)
Tetracyclines						
Oxytetracycline	2 (25)	5 (62.5)	3 (50)	1 (33.33)	0	1 (100)
Penicillins						
Ampicillin	1 (12.5)	6 (75)	2 (33.33)	0	0	0%
Amoxicillin	1 (12.5)	4 (50)	2 (33.33)	1 (33.33)	0	0%
Cloxacillin	1 (12.5)	4 (50)	2 (33.33)	0	0	1 (100)
Penicillin	1 (12.5)	6 (75)	2 (33.33)	0	0	0
Amoxicillin/clavulanic acid	3 (37.5)	7 (87.5)	3 (50)	2 (66.67)	0	0
Fluoroquinolones						
Enrofloxacin	2 (25)	3 (37.5)	3 (50)	1 (33.33)	1 (100)	0
Aminoglycosides						
Gentamicin	6 (75)	4 (50)	4 (66.67)	3 (100)	1 (100)	1 (100)
Amikacin	6 (75)	1 (12.5)	3 (50)	1 (33.33)	1 (100)	1 (100)
Neomycin	7 (87.5)	1 (12.5)	4 (66.67)	1 (33.33)	1 (100)	0
Streptomycin	4 (50)	2 (25)	3 (50)	1 (33.33)	1 (100)	0
Cephalosporins						
Ceftriaxone	5 (62.5)	8 (100)	3 (50)	3 (100)	1 (100)	1 (100)
Cefoperazone	5 (62.5)	8 (100)	3 (50)	1 (33.33)	1 (100)	1 (100)
Ceftriaxone/tazobactam	6 (75)	8 (100)	5 (83.33)	3 (100)	1 (100)	1 (100)
Macrolides						
Chloramphenicol	6 (75)	8 (100)	3 (50)	2 (66.67)	0	1 (100)

E. coli=Escherichia coli

In case of *Streptococcus* spp., the maximum sensitivity 100% was seen toward ceftriaxone, cefoperazone, chloramphenicol, and ceftriaxone-tazobactam followed by amoxicillin-clavulanic acid (87.5%), penicillin-G and ampicillin (75%), oxytetracycline (62.5%), gentamicin, amoxicillin and cloxacillin (50%), enrofloxacin (37.5%), and streptomycin (25%). Amikacin and neomycin (12.5%) were found to be least sensitive (Table-3).

Maximum sensitivity of *Staphylococcus* spp. was observed for ceftriaxone-tazobactam (83.33%), followed by gentamicin and neomycin (66.67%), chloramphenicol, ceftriaxone, cefoperazone, streptomycin, oxytetracycline, amoxicillin-clavulanic acid, enrofloxacin, and amikacin (50%) and least toward amoxicillin, ampicillin, cloxacillin, and penicillin-G (33.33%) (Table-3). *Klebsiella* spp. showed maximum sensitivity toward ceftriaxone-tazobactam, gentamicin, and ceftriaxone (100%) followed by chloramphenicol and amoxicillin-clavulanic acid (66.67%), neomycin, amikacin, cefoperazone, streptomycin, oxytetracycline, enrofloxacin, and amoxicillin (33.33%) (Table-3).

*Pseudomonas* spp. obtained in the present study was found to be sensitive toward enrofloxacin, gentamicin, amikacin, neomycin, streptomycin, ceftriaxone, cefoperazone, and ceftriaxone/tazobactam combination and resistant toward chloramphenicol, oxytetracycline, ampicillin, amoxicillin, cloxacillin, penicillin, and amoxicillin/clavulanic acid (Table-3).*Bacillus* spp. isolate was sensitive toward ceftriaxone, cefoperazone, ceftriaxone/tazobactam, chloramphenicol, gentamicin, amikacin, oxytetracycline, and cloxacillin and resistant toward ampicillin, amoxicillin, penicillin-G, amoxicillin/clavulanic acid, enrofloxacin, neomycin, and streptomycin (Table-3).

### Determination of MDR isolates

On the phenotypic *in* *vitro* sensitivity basis, we found 24 isolates to be MDR, i.e., resistant to three or more than three antibiotics belonging to different antibiotic groups. Among these MDR bacteria, we found two isolates to be XDR, whereas one was found to be pandrug-resistant isolate as shown in Table-4.

**Table-4:** Determination of multidrugresistant isolates using* in vitro* sensitivity.

Bacterial isolates			1	2	3	4	5	6	7	8	9	10	11	12	13	14	15	16	17	18	19	20	21	22	23	24	25	26	27
Antibiotics (1-15) within antibiotic groups (A-G)	A	1	R	R		R	R	R	R	R	R	R					R	R	R			R	R		R	R			RR
	B	2	R	R				R		R			R				R	R	R	R	R	R	R	R	R	R			R
		3	R	R		R	R	R	R			R	RR				R	R	R	R	R	R	R	R	R	R			R
		4	R	R		R	R	R	R	R		R						R	R	R	R	R	R		R	R	R	R	R
		5	R	R		R	R	R		R			R				R	R	R	R	R	R	R	R	R	R			R
	C	6	R	R		R	R										R		R			R	R	R	R	R			R
	D	7	R	R		R	R				R		R	R	R	R		R	R	R	R	R	R	R					R
	E	8	R	R					R			R		R	R	R						R							
		9	R	R		R	R		R		R	R		R	R	R		R				R					R	R	
		10	R	R		R	R		R	R	R	R	R	R	R	R								R					
		11	R	R		R	R		R	R		R	R	R	R	R			R	R	R	R		R					
	F	12	R	R															R	R	R	R							
		13	R	R						R R								R	R	R	R	R							
		14		R															R			R							
	G	15	R	R						R							R					R			R				

(A) Tetracyclines (1-Oxytetracycline), (B) Penicillins (2-ampicillin; 3-amoxycillin; 4-cloxacillin; 5-penicillin), (C) Penicillin/β lactamase inhibitor combination (6-amoxycillin/clavulanic acid), (D) Fluoroquinolones (7-enrofloxacin), (E) Aminoglycosides (8-gentamicin; 9-amikacin; 10-neomycin; 11-streptomycin), (F) Cephalosporins (12-ceftriaxone; 13-cefoperazone; 14-ceftriaxone/tazobactum), (G) Macrolides (15-chloramphenicol). R=Resistant, Blank=Sensitive

## Discussion

UTI is thought to be the most common infectious disease of dogs. In the present study, 35 urine samples were inoculated on different media, and 22 samples were found positive on culture examination which is considered as a definitive diagnostic test. Six different genera of bacteria were isolated. Among the various microorganisms isolated, *E. coli* (29.62%) and *Streptococcus* spp. (29.62%) were the most prevalent followed by *Staphylococcus* spp. (22.22%), *Klebsiella* spp. (11.11%), *Pseudomonas* spp. (3.7%), and *Bacillus* spp. (3.7%). Our findings of *E. coli* as the most prevalent bacteria associated with UTI in dogs have been strengthened by some researchers [3,5,6,10-16].

Selection of suitable antimicrobial for treatment primarily depends on the sensitivity of the organism isolated. In the present study, large numbers of isolates were found to be resistant to a different group of antibiotics. This resistance can be attributed to the indiscriminate use of antibiotics against bacteria resulting in the emergence of drug-resistant mutants which often leads to treatment failure in dogs. Overall, *in* *vitro* antibiotic sensitivity revealed cephalosporins and macrolides as the most sensitive and penicillins showed the least sensitivity toward all the isolates.

Of all the antibiotics tested for the determination of *in* *vitro* sensitivity of *E. coli* isolates, aminoglycosides outstood with the range of 50-87.5%. Among aminoglycosides, neomycin was found to be most effective with 87.5% sensitivity. This can be attributed to lesser use of these antibiotics due to nephrotoxic nature of these drugs. Least sensitivity of *E. coli* was seen toward penicillin group, especially ampicillin, amoxicillin, cloxacillin, and penicillin-G while higher sensitivity of *E. coli* toward penicillin drugs has been reported by other researchers [14,16,17]. The best possible reason described for this difference could be due to routine use of different antibiotics in different area and difference in management practices might be leading to the generation of mutational strain consequently resistant bacteria.

Results of antibiotic sensitivity pattern for *Streptococcus* spp. showed 100% sensitivity toward cephalosporins and chloramphenicol, 87.5% isolates sensitive toward amoxicillin/clavulanic acid combination, and 62.5% sensitive toward tetracycline. Similar results of sensitivity toward amoxicillin-clavulanic acid and tetracycline have been reported by Windahl *et al*. [14]. In the present study, 75% *Streptococcus* spp. isolates were found sensitive to penicillin group of antibiotic in which was contrary to the findings of Windahl *et al*. [14] showing 100% sensitivity. The probable reason for the development of resistance by this organism to penicillin in Indian condition is conventional, prolonged, and indiscriminate use of this drug in the field conditions. *Staphylococcus* spp. has been found most sensitive (83.33%) toward ceftriaxone/tazobactam combination and least (33.33%) toward penicillin drugs. The resistance of *S. aureus* to penicillin group of antibiotics in our study which is due to the production of beta-lactamase, an enzyme that inactivates penicillin and closely related antibiotics.

*Klebsiella* isolates have been found 100% sensitive toward gentamicin and ampicillin which was in accordance with the findings of Kogika *et al*. [11]. 100% resistance of *Klebsiella* isolates toward ampicillin, cloxacillin, and penicillin in the present study is in accordance with the findings of Windahl *et al*. [14] showing complete resistance toward ampicillin. This resistant behavior of *Klebsiella* isolates toward ampicillin and other penicillins can be attributed to the production of β-lactamase enzyme destroying the antibiotics.

These findings of variable sensitivity of bacteria isolated toward different antimicrobial suggest that the antimicrobial agent to be used should be selected on the basis of bacterial culture and results of antibiotic sensitivity tests and clinical response to the antibiotic.

## Conclusion

The present study indicated considerable resistance in pathogens associated with UTI in dogs. *E. coli* and *Streptococcus* spp. were the most prevalent bacteria isolated from UTI affected dogs. *In vitro* antimicrobial sensitivity testing revealed cephalosporins and macrolide group to be the most effective group of antibiotics.

## Authors’ Contributions

AK supervised the whole study. MP and AK designed the study. MP and TK performed the cultural examination and determination of sensitivity. GC and MP analyzed the data and wrote the manuscript. The final manuscript has been read and approved by all the authors.
